# Natamycin-Loaded Ethyl Cellulose/PVP Films Developed by Microfluidic Spinning for Active Packaging

**DOI:** 10.3390/foods13010132

**Published:** 2023-12-29

**Authors:** Xiangzheng Yang, Jingshan Rao, Chaoyi Shen, Huan Lian, Da Wang, Di Wu, Kunsong Chen

**Affiliations:** 1 Zhejiang Provincial Key Laboratory of Horticultural Plant Integrative Biology, Key Laboratory of Ministry of Agriculture and Rural Affairs of Biology and Genetic Improvement of Horticultural Crops (Growth and Development), College of Agriculture and Biotechnology, Zhejiang University, Hangzhou 310058, China; yangxiangzheng318@163.com (X.Y.); 22016039@zju.edu.cn (J.R.); wangda19910@163.com (D.W.); akun@zju.edu.cn (K.C.); 2 Jinan Fruit Research Institute, All China Federation of Supply and Marketing Cooperatives, Jinan 250014, China; lianh_jy@163.com; 3 College of Biosystems Engineering and Food Science, Zhejiang University, Hangzhou 310058, China; 21916123@zju.edu.cn

**Keywords:** microfluidic spinning, ethyl cellulose, polyvinylpyrrolidone, natamycin, antimicrobial activity

## Abstract

The preparation of active packaging loaded with antimicrobial, antioxidant, and other functional agents has become a hot topic for food preservation in recent years. In this field, active fiber films based on spinning methods have attracted the interest of researchers owing to their high specific surface area, high porosity, high loading capacity, and good controlled release capacity. In the present work, neatly arranged ethyl cellulose (EC)/polyvinyl-pyrrolidone (PVP) fibrous films loaded with natamycin as an antimicrobial agent were prepared by microfluidic spinning. The encapsulation efficiency of natamycin was more than 90% in each group and the loading increased with increasing natamycin content. According to the characterization results of the natamycin-loaded EC/PVP fibrous films, hydrogen bonding was formed between natamycin and EC and PVP in the fibrous films. Meanwhile, the water contact angle of the fibrous films was increased, suggesting the improved hydrophobicity of the films. In the in vitro bacterial inhibition experiments, the active fiber films loaded with natamycin showed good antimicrobial activity, which could significantly inhibit the growth of gray mold. In conclusion, N-EC/PVP fibrous films with antimicrobial activity prepared by microfluidic spinning showed good potential in the field of active packaging.

## 1. Introduction

Postharvest fruits and vegetables deterioration and decay are important factors affecting the sustainable development of the fruits and vegetables industry [1,2,3]. Fungal infections are one of the main causes of fruit deterioration and decay [4]. Among them, gray mold is a widespread fungus that can not only infect crops in the field, but also cause huge losses to the post-harvest stage of fruits and vegetables [5]. Gray mold can infect various fruits and vegetables, causing them to rot after harvest and causing serious economic losses, such as strawberries [6], tomatoes [7], grapes [8]. Therefore, it is necessary to effectively control the infection of gray mold in the post-harvest stage of fruits and vegetables.

Natamycin is a type of polyene macrolide polyketide acid, one of the most efficient fruit preservatives, widely utilized in food processing and recognized by the European Food Safety Agency and the U.S. Food and Drug Administration [9]. Natamycin specifically binds to ergosterol to inactivate yeast and mold, but does not penetrate the plasma membranes. It inhibits the growth of various fungi and the production of very low concentrations of fungal toxins [10]. However, the low water solubility and photosensitivity of natamycin result in its high cost and low utilization, which limits its application in postharvest fruit [11]. There have been several studies to encapsulate natamycin to improve its utilization efficiency. Fang et al. [12] synthesized gliadin/carboxymethyl chitosan nanoparticles to encapsulate natamycin, which enhanced the photostability of natamycin. In another work, Yuan et al. [13] prepared sodium alginate/gum arabic/glycerol film containing natamycin and successfully applied it to sweet potato preservation. The results showed that the prepared film delayed the decline in the water content, total starch content, Vc content, and flavonoid glycoside content of sweet potato during the storage period, and maintained the processing quality of sweet potato even after 120 days [13].

In recent years, the creation of active packaging with functions such as preservation, and sterilization has received widespread attention [14,15]. In particular, fibrous films composed of micro- and nanoscale fibers are considered to be good carriers for loading active substances, owing to their high specific surface area, high porosity, and other advantages [16,17]. As a commonly used method for preparing fresh-keeping fiber films, electrospinning technology has been chosen by a large number of researchers due to its early start and mature technical system [18,19]. However, the electrospinning preparation process requires high-voltage electrostatic driving, which not only poses certain safety hazards, but also puts forward high requirements for the conductivity of the spinning solution [20]. Microfluid spinning technology is an emerging method for preparing fibrous films. It mainly produces ordered arranged fibers through physical/chemical crosslinking or direct solidification of polymer solutions at the exit of microchannels, which has the advantages of simple operation, low cost, and controllable fiber morphology and structure [21,22]. Lin et al. [23] developed chlorogenic acid-loaded poly(methyl methacrylate)/konjac glucomannan films using microfluidic spinning, which exhibited good antibacterial activity. In another work, Zhang et al. [24] fabricated ampicillin-incorporated alginate/chitosan fibers by microfluidic spinning, which also exhibited good antibacterial activity.

Ethyl cellulose (EC), a water-insoluble cellulose derivative, is one of the very important hydrophobic nonionic polysaccharides, which has been extensively applied in the food and pharmaceutical industries due to its unique properties of naturalness, biocompatibility, and biodegradability [25,26]. In addition, EC has good film-forming properties, mechanical properties, thermal stability and hydrophobicity, which makes it an excellent candidate for film-forming materials [25]. Rashidi et al. [27] prepared EC and soy protein-based fibers containing bitter orange peel extract using electrospinning and used them in active packaging, which showed good antimicrobial activity and antioxidant activity. In another work, Beikzadeh et al. [28] developed EC/poly caprolactone/gelatin fibers containing Zataria multiflora essential oil by electrospinning, which exhibited good mechanical properties, antifungal activity, and antioxidant activity. Polyvinylpyrrolidone (PVP) is an important class of amorphous polymers characterized by low chemical toxicity, high biocompatibility, good solvency, and high spinnability [29]. PVP is also frequently selected as a substrate material to prepare films and for applications in active food packaging. Taher et al. [30] prepared edible coating films for tomato fruit storage using PVP and gum arabic, and the results showed that these films could delay fruit ripening and maintain the antioxidant capacity of tomato fruit.

In our previous research, micron sized EC/PVP fibrous fibers were successfully prepared through microfluidic spinning [21]. However, the materials prepared in the previous study were not loaded with active substances and therefore lacked antimicrobial properties. In the present study, natamycin was selected as the active substance, and was encapsulated into the substrate material during the spinning process. EC and PVP were used as substrate materials, and microfluidic spinning technology was used to prepare arranged fibrous films loaded with natamycin at room temperature and pressure. SEM, FTIR, XRD, and water contact angle measurement were utilized to analyze the changes in various physical and chemical properties of the EC/PVP fiber film after loading natamycin, and its antibacterial ability was evaluated.

## 2. Materials and Methods

### 2.1. Materials

Polyvinylpyrrolidone (PVP, K88-96, molecular weight 1.3 million) and ethyl cellulose (EC, 3–7 mPa s, molecular weight 20–30 kDa) were provided by Aladdin Reagent Co., Ltd. (Shanghai, China). Anhydrous ethanol (analytical grade) was provided by China National Pharmaceutical Group Chemical Reagent Co., Ltd. (Shanghai, China). Natamycin (95% purity) was purchased from Aladdin Reagent Co., Ltd. (Shanghai, China). The strain of *Botrytis cinerea* was purchased from the Shanghai Biological Preservation Technology Center (Shanghai, China).

### 2.2. Preparation of Active Fibrous Films by Microfluidic Spinning

According to the previous research [21], EC and PVP were selected to prepare fibrous films in a ratio of 2:3, where 1.6 g EC and 2.4 g PVP were solubilized in 20 mL of 90% ethanol. Subsequently, Natamycin (0.02 g, 0.04 g, 0.1 g, and 0.2 g) was added to the EC/PVP solution to obtain N-EC/PVP solutions with different concentrations of natamycin as precursor solutions for microfluidic spinning.

A microfluidic air jet spinning machine (JNS- MS-SBS-01, Nanjing Janus New Materials Co., Ltd., Nanjing, China) was utilized to conduct microfluidic spinning process. Every spinning solution was injected into a 20 mL syringe, which was connected to the needle via silicone tubing with an inner and outer diameter of 3 mm and 4 mm, respectively, and secured to a microfluidic syringe pump. The solution flow rate was set to 0.6 mL/h, and a rectangular plastic frame connected to a motor with a rotational rate of 500 rads/min was used as a receiver to collect fibers, and the horizontal movement speed of the needle is set to 1 cm/min to obtain an orderly arranged fiber film of appropriate width. The fiber films prepared based on EC/PVP solution and N-EC/PVP solution with different concentrations of Natamycin were named Nata 0, Nata 0.02, Nata 0.04, Nata 0.1, and Nata 0.2, respectively.

### 2.3. Encapsulation Efficiency and Loading Capacity

An absorbance method was utilized to measure the encapsulation efficiency and loading capacity of the natamycin within the EC/PVP fibers. The 5 mg samples were put into 10 mL of 90% ethanol under shaking until the samples were completely dissolved. A microplate reader (Tecan Infinite M200 Pro, Mannedorf, Switzerland) was utilized to determine the encapsulation efficiency and loading capacity of natamycin by recording the absorbance at 303 nm.

### 2.4. Morphology

The microstructure of the natamycin-loaded EC/PVP fiber morphology was investigated utilizing a scanning electron microscope (SU8010, Hitachi Corporation Hitachi Production Office, Tokyo, Japan). Before SEM observation, all samples were subjected to surface gold spraying treatment. Using Image J software (Version 1.2), 100 fibers were randomly selected from the scanning electron microscope images of the fibrous films and their average diameters were measured, which were then used as the fiber diameters of the fibrous film samples.

### 2.5. FTIR Assay

A small number of fiber films and potassium bromide particles were thoroughly grinded, and then the samples were analyzed using an infrared spectrometer (Nicolet iS50, Thermo Scientific, Waltham, MA, USA). The infrared spectral signatures of the samples were measured in the wavelength region of 4000 to 400 cm^−1^, with a scanning resolution of 4 cm^−1^, a total of 32 times [29].

### 2.6. Crystal Structure Assay

The X-ray diffraction patterns of each sample were investigated utilizing an X-ray diffractometer (Bruker AXS D8 Advance, Bruker Corporation, Saarbrücken, Germany), with a diffraction region of 5–90° (2θ), and a scanning speed of 2°/min [31].

### 2.7. Surface Wettability Assay

The water contact angle testing on samples was conducted utilizing a video optical contact angle measuring machine (OCA 20, Dataphysics Corporation, Stuttgart, Germany). The fiber film samples were fixed on a glass slide, deionized water was utilized as the probe solvent, and 3 μL of deionized water was dropped onto the flat surface of the samples. And after 10 s of equilibrium, the water contact angles on both sides were measured. Every sample was measured three times and the average value was taken.

### 2.8. Antimicrobial Activity Assay

The samples were cut into several small circular pieces with a diameter of 10 mm and then were sterilized on both sides under ultraviolet light for 1 h (30 min on each side).

The strain of *Botrytis cinerea* was stored in a −80 °C refrigerator before use. The strain was inoculated onto a PDA plate, cultured upside down in a 25 °C incubator, and reincubated at 7 d intervals. In a sterile environment, 5 mL of purified water was applied to a PDA plate filled with *Botrytis cinerea* spores, the surface of the plate was gently scraped with an applicator stick to suspend the spores repeatedly, and then was prepared at a concentration of about 10^6^ CFU/mL by constant dilution using a blood cell counter. Then, 100 μL of suspension of *Botrytis cinerea* was spread uniformly on PDA medium, and then the sterilized fibrous film discs were put in the center of the PDA medium. They were inverted and cultured in a 25 °C incubator, and the inhibitory effect was observed and recorded from the central inhibitory area.

### 2.9. Statistical Analysis

Single factor analysis of variance (ANOVA) and Duncan multiple comparison experiment were conducted using SPSS v.26.0 statistical software (IBM, Armonk, NY, USA). When *p* < 0.05, the results were considered to have significant differences. Origin Pro 2019 version (OriginLab, Northampton, MA, USA) was used for drawing.

## 3. Results

### 3.1. Encapsulation Efficiency, Loading Capacity, and Fiber Morphology

The results of encapsulation efficiency and loading capacity of the samples with various weights of natamycin are shown in Table 1. The results show that the encapsulation efficiency of natamycin was 95.03%, 93.44%, 92.60%, and 90.20% in Nata 0.02, Nata 0.04, Nata 0.1, and Nata 0.2, respectively. The encapsulation efficiency of natamycin in all fibers was more than 90%, which proves that microfluidic spinning is a method with high encapsulation efficiency. In addition, the loading efficiency of fibers on natamycin increased with increasing natamycin content. Specifically, the loading efficiency of natamycin was 4.75‰, 9.34‰, 23.17‰, and 45.10‰ in Nata 0.02, Nata 0.04, Nata 0.1, and Nata 0.2, respectively.

**Figure 1:**
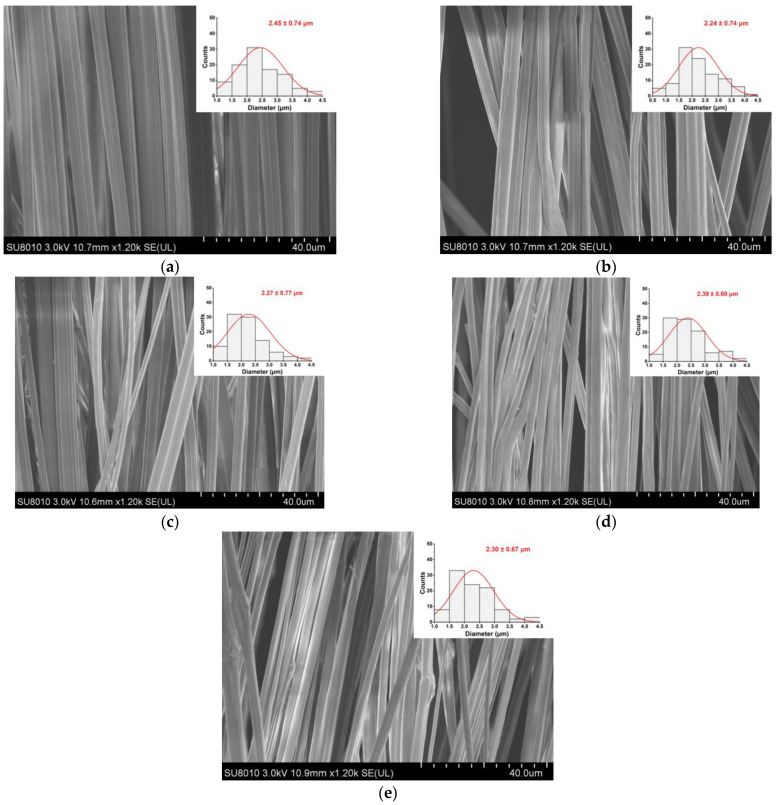
SEM images of the films. (**a**) Nata 0; (**b**) Nata 0.02; (**c**) Nata 0.04; (**d**) Nata 0.1; (**e**) Nata 0.2.

As shown in Figure 1, the fiber morphology and diameter distribution of the EC/PVP and N-EC/PVP fibrous films loaded with different natamycin (Nata 0, Nata 0.02, Nata 0.04, Nata 0.1, Nata 0.2) were observed by SEM. The fibers of all the samples showed an oriented and well-ordered structure. It could be seen that the addition of natamycin did not have a significant impact on the normal preparation of the fibers.

One hundred fibers from each group of fiber films were randomly selected for diameter measurement, which were 2.45 ± 0.74 µm (Nata 0), 2.24 ± 0.74 µm (Nata 0.02), 2.27 ± 0.77 µm (Nata 0.04), 2.39 ± 0.69 µm (Nata 0.1), and 2.30 ± 0.67 µm (Nata 0.2), respectively. It could be seen that there was no remarkable difference in fiber diameter among these groups, basically around 2.30 µm, and there was no significant correlation with the concentration of natamycin added. This may be because natamycin was a small molecule substance compared to the EC and PVP polymers in the spinning solution, and the amount added was relatively small in this experiment. It was evenly dispersed in the solution after stirring, so the effect on the viscosity of the solution was almost negligible.

### 3.2. FTIR

**Figure 2:**
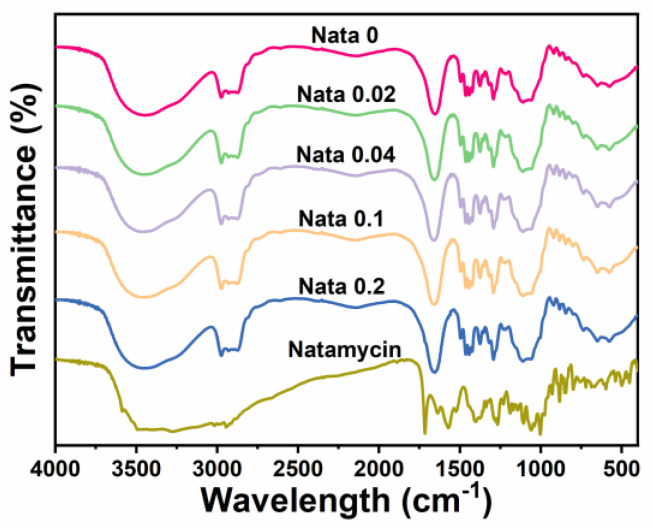
FTIR spectra of the natamycin-loaded fibrous films and natamycin.

FTIR analysis (4000 cm^−1^ to 400 cm^−1^) was performed on the EC/PVP and N-EC/PVP fibrous films, and the results are shown in Figure 2. It could be seen that the overall infrared spectrum curves of each sample were basically similar, indicating that EC and PVP in the prepared fibrous film were uniformly mixed. In each group of FTIR curves, some characteristic peaks belonging to EC and PVP can be observed, such as the stretching vibration of C=O in 1655 cm^−1^ (PVP) [32], and larger broad peaks in the wavelength range of 3750 cm^−1^ to 3000 cm^−1^ (stretching vibration of O−H bonds).

Natamycin also displayed a large absorption peak in the range of 3750 cm^−1^ to 3000 cm^−1^, owing to the stretching vibration of the O-H and N−H bonds in its structure. Other characteristic peaks of natamycin can also be recorded in the graph, such as the absorption peak near 1715 cm^−1^ (C=O), the absorption peak near 1267 cm^−1^ (C−O−C), the absorption peak near 1105 cm^−1^ (C−OH asymmetric stretching), and the absorption peak near 1002 cm^−1^ (C−H deformation in CH=CH) [33]. However, the characteristic peaks belonging to Natamycin mentioned above were not observed in the absorption spectrum curve of the fibrous film, which may be due to the low content of Natamycin added to the fiber film [9]. However, by comparing the absorption peak intensities of each group, it could be found that the absorption peak intensities of N-EC/PVP fiber film were strengthened in certain bands, such as the absorption peak intensity near 1655 cm^−1^ exceeding Nata 0, which may be caused by the addition of Natamycin.

### 3.3. XRD

**Figure 3:**
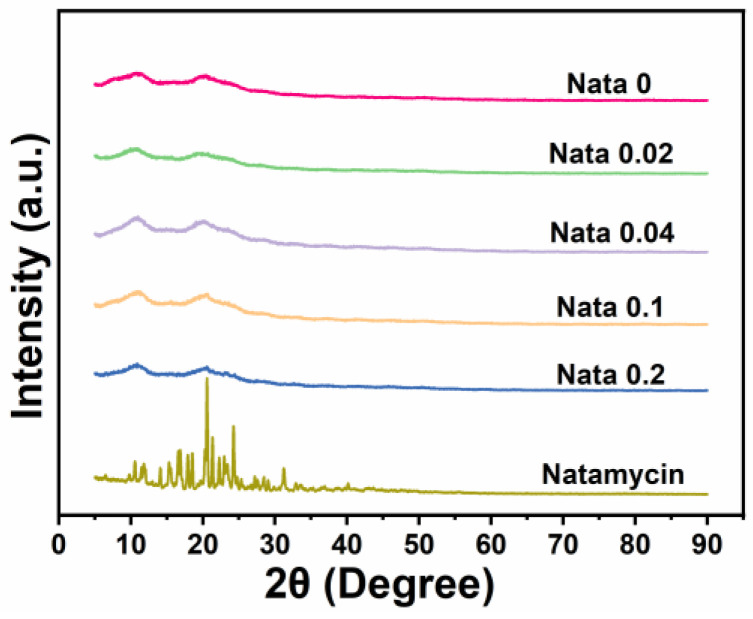
XRD patterns of the films and natamycin.

Figure 3 displays the X-ray diffraction patterns of the natamycin-loaded EC/PVP fibrous films. Natamycin, as a crystal, exhibited many characteristic diffraction peaks in the range of 10–30°, especially sharp peaks near 20.5°, indicating that natamycin is highly crystalline [34]. For different fiber films (Nata 0, Nata 0.02, Nata 0.04, Nata 0.1, and Nata 0.2), two smaller and wider diffraction peaks could be observed near 10.9° and 20.5°. This was mainly attributed to the XRD pattern of EC, which has two peaks at 7.9° and 20.2°. Tommalieh et al. [35] found that PVP has two wider diffraction peaks at 11.8° and 20.8°. Therefore, for each EC/PVP fiber film prepared from EC and PVP, corresponding diffraction peaks were observed around 10.9° and 20.5°. In addition, no characteristic peaks of natamycin were recorded in the diffraction patterns of Nata 0.02, Nata 0.04, Nata 0.1, and Nata 0.2, and no significant differences in diffraction peak intensity were observed in each diffraction curve. This may be due to the low concentration of natamycin added [36].

### 3.4. Surface Wettability Analysis

**Figure 4:**
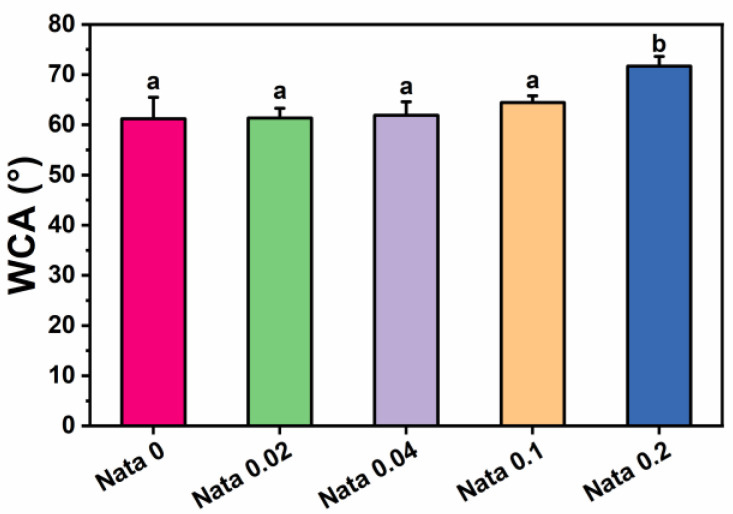
Water contact angle (WCA) of the natamycin-loaded EC/PVP films. Different letters for each sample indicate significant differences (*p* < 0.05).

Water contact angle tests were carried out on the natamycin-loaded EC/PVP fibrous films to investigate the effect of the incorporation of natamycin on the surface hydrophilicity of fibrous films. The test results of water contact angle measurements are presented in Figure 4. The water contact angle data of the fibrous films of each group at 10 s were 61.2 ± 4.3° (Nata 0), 61.4 ± 1.9° (Nata 0.02), 61.9 ± 2.7° (Nata 0.04), 64.4 ± 1.4° (Nata 0.1), and 71.7 ± 1.9° (Nata 0.2), respectively. It could be seen that as the content of natamycin in the fiber film increased, the water contact angle displayed an upward trend. When the content of natamycin was low, the water contact angle of the fiber film changed relatively insignificantly. Also, the significance analysis results showed that there was no remarkable difference between the water contact angles of Nata 0, Nata 0.02, Nata 0.04, and Nata 0.1, especially the water contact angles of the first three groups were basically similar. The water contact angle of Nata 0.2 was significantly higher than other groups, exceeding 70°. This was mainly because natamycin contains hydrophobic groups with double bond structures in macrolides, which were almost insoluble in water [37]. Therefore, it has good hydrophobicity, resulting in better hydrophobicity of fiber films with higher natamycin content. In addition, the fibrous film prepared by adding natamycin to the EC/PVP spinning solution also had a small amount of natamycin on its surface, which would also play a certain role in blocking water. The above results suggested that the addition of natamycin effectively enhanced the surface properties of N-EC/PVP fibrous films, including reduced water absorption and further increased hydrophobicity. Usually, the storage environment of fruits and vegetables has high humidity, and materials with poor hydrophobicity are prone to structural collapse during storage, which leads to failure of the active packaging. The hydrophobic surface allows packaging materials to be utilized in high humidity environments and reduces microbial adhesion [38].

### 3.5. Antimicrobial Activity Assay

**Figure 5:**
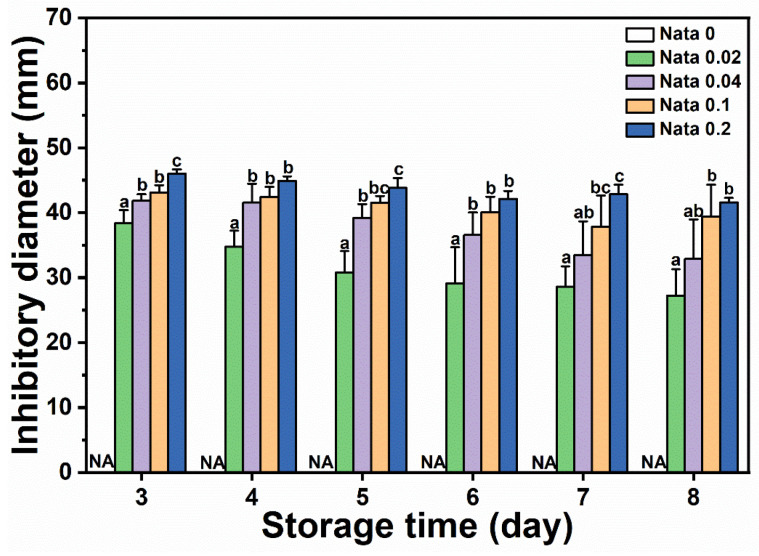
Inhibitory diameters of *Botrytis cinerea* of the films. Different letters for each sample indicate significant differences (*p* < 0.05).

N-EC/PVP fibrous film discs loaded with different concentrations of natamycin were placed in the center of a culture dish coated with *Botrytis cinerea* suspension, and the diameter of the inhibitory zone on the culture dishes of different treatment groups was measured and recorded from the first day when mycelium germination can be clearly observed. The results are shown in Figure 5. It could be seen that the EC/PVP fibrous film (Nata 0) without natamycin did not have antimicrobial activity, while the active fibrous film loaded with natamycin had significant antimicrobial effect. The diameters of the inhibitory zones in each group were 38.4 ± 2.0 mm (Nata 0.02), 41.8 ± 1.0 mm (Nata 0.04), 43.1 ± 1.1 mm (Nata 0.1), and 46.0 ± 0.7 mm (Nata 0.2), respectively, and showed an overall trend of increasing with the increase in natamycin content. However, compared to Nata 0.02, although Nata 0.04, Nata 0.1, and Nata 0.2 loaded more natamycin, the increase in the diameter of their inhibitory zones was not significant. Based on a comprehensive analysis of the amount of natamycin added and the antibacterial effect of the fibrous film, it was believed that Nata 0.02 exhibited superior antibacterial properties in the early stages. At the same time, it also indicated that natamycin had good antimicrobial activity, which could exert antimicrobial effects at lower concentrations, similar to the results of González and Alvarez Igarzabal [39]. In a previous work, Mo et al. [40] prepared gelatin films containing natamycin-loaded zein/casein nanoparticles, which showed an inhibitory diameter of about 35 mm against *Botrytis cinerea*. In conclusion, the fibrous films obtained by microfluidic spinning in the present study has a good inhibitory effect on *Botrytis cinerea*. In our previous work, gelatin/zein/polyurethane nanofibers loaded with natamycin were prepared via the solution blow spinning technique, exhibiting inhibitory diameters of 22–70 mm against *Botrytis cinerea* [9]. The fibrous films in the present study had inhibitory diameters of 27–50 mm and possessed antifungal activity comparable to that of nanofibers prepared by solution blow spinning technique.

On the other hand, as *Botrytis cinerea* spores grew, the antimicrobial zone gradually decreased. On the 8th day, the diameter of the inhibitory zone in each group decreased to 27.3 ± 4.0 mm (Nata 0.02), 32.9 ± 6.0 mm (Nata 0.04), 39.4 ± 4.9 mm (Nata 0.1), and 41.6 ± 0.7 mm (Nata 0.2), respectively. However, it should be noted that the diameter of the antimicrobial zone of the fibrous film loaded with a low concentration of natamycin decreases rapidly, while the diameter of the antimicrobial zone of the fibrous film loaded with a high concentration of natamycin could be well maintained (the antimicrobial diameter of the Nata 0.2 fibrous film still exceeded 40 mm on the 8th day), demonstrating long-lasting antimicrobial ability. This also indicated that although the antimicrobial cost-effectiveness of fibrous films with a higher content of natamycin was not high (the concentration difference between Nata 0.02 and Nata 0.2 fiber films loaded with natamycin was an order of magnitude, but the difference in the diameter of the inhibitory zone between the two was relatively small) in the early stage (especially on the 3rd day), fibrous films loaded with higher concentrations of natamycin still had a higher cost-effectiveness in terms of antibacterial effect on *Botrytis cinerea* in the later stage.

The fibrous films in this work exhibited good antifungal activity against Botrytis cinerea. Therefore, the fibrous films are suitable for applications with some fruits and vegetables that are susceptible to gray mold, such as cucumbers, strawberries, and tomatoes.

In our study, the lowest amount of natamycin (0.02 g) demonstrated good antifungal activity. In future practical applications, we will try to further reduce the amount of natamycin used, taking into account the cost of preparation and ensuring the antimicrobial effect.

In practical applications, the fibrous films can be used as liners in commercial packaging materials to achieve antifungal effects by contact with fruits and vegetables. Additionally, by replacing the rectangular plastic frame (the receiver for fibers) with fruits and vegetables, it is possible to deposit fibers directly on the surface of fruits and vegetables. This is what our research group has been working on recently. In all the above application scenarios, the mechanical properties of the fibrous films are not very demanding, so the mechanical properties of the material are not considered in this work. However, in future research and practical applications, we will consider the mechanical properties of the fibrous films. At the same time, research on the mechanical properties of fibrous films should be conducted in future when faced with application scenarios that require enhanced mechanical properties.

## 4. Conclusions

In this research, natamycin-loaded EC/PVP fibrous films were prepared using microfluidic spinning technology. The SEM and FTIR results showed that natamycin was successfully encapsulated into EC/PVP fibers without affecting the morphology of the fibers. The encapsulation efficiency of natamycin was more than 90% in each group and the loading increased with increasing natamycin content. Due to the low content of natamycin added, no crystallization peak of natamycin was observed in the XRD patterns. With the incorporation of natamycin, the water contact angle of EC/PVP increased, indicating its improved hydrophobicity. The N-EC/PVP fibrous film exhibited excellent antimicrobial activity against *Botrytis cinerea*. The above results indicated that the N-EC/PVP fibrous film has good application prospects in postharvest antimicrobials and the preservation of fruits and vegetables.

## Figures and Tables

**Table 1:** Encapsulation efficiency and loading capacity of the nanofibers with various weights of encapsulated natamycin.

Sample	Encapsulation Efficiency (%)	Loading Capacity (‰)
Nata 0.02	95.03 ± 1.27	4.75 ± 0.06
Nata 0.04	93.44 ± 1.62	9.34 ± 0.16
Nata 0.1	92.60 ± 1.08	23.17 ± 0.27
Nata 0.2	90.20 ± 0.65	45.10 ± 0.33

## Data Availability

Data is contained within the article.
